# Age differences in selected measures of physical fitness in young handball players

**DOI:** 10.1371/journal.pone.0242385

**Published:** 2020-11-12

**Authors:** Jaime Fernandez-Fernandez, Isidoro Martinez-Martin, Vicente Garcia-Tormo, Juan Garcia-Lopez, Mario Centeno-Esteban, Lucas A. Pereira, Irineu Loturco

**Affiliations:** 1 Faculty of Physical Activity and Sports Sciences, Universidad de León, León, Spain; 2 AMRED, Human Movement and Sports Performance Analysis, Universidad de León, León, Spain; 3 NAR – Nucleus of High Performance in Sport, São Paulo, Brazil; 4 Department of Human Movement Sciences, Federal University of São Paulo, São Paulo, Brazil; 5 University of South Wales, Pontypridd, Wales, United Kingdom; Universidade Federal de Mato Grosso do Sul, BRAZIL

## Abstract

**Objective:**

The aims of the present study were: 1) to calculate the change of direction (COD) deficit (using a modified version of the 505 test and 10 m sprint time), and (2) to examine the differences in linear sprint, jump and COD performances, as well as COD deficit, between under-13 (U13) and under-15 (U15) male handball players.

**Methods:**

One hundred and nineteen young male handball players (under-13 [U13; n = 82] and under-15 [U15; n = 37]). Tests included anthropometric measurements, countermovement jump (CMJ), triple leg-hop for distance, linear sprint test (5, 10 and 20 m), and a modified version of the 505 COD test.

**Results:**

Results showed moderate to very large differences (*P* < 0.05) in age, predicted age at peak height velocity (APHV), distance from PHV (DPHV), height, and body mass between the age categories. Moreover, U15 players demonstrated higher performances in all jump tests and lower sprint times in 10- (ES = 0.84) and 20-m (ES = 0.51) and a higher 505 deficit (ES = 0.38) than the U13 players (*P* < 0.05). However, no significant differences were observed for the 505 COD test between groups (ES = 0.12; *P* > 0.05).

**Conclusions:**

Our results suggest that during the transition from pre- to post-puberty, young handball players should focus on transferring their progressive improvements in strength, speed, and power capacities to COD performance.

## Introduction

Handball is an intermittent sport, which requires good levels of different physical qualities, such as endurance, strength, speed, and coordination [1]. In terms of performance, handball is characterized by several high-intensity actions during matches, including jumps, accelerations, decelerations, and changes of direction (COD) [2]. In this regard, during competitions, handball players may perform more than 30 COD tasks and cover up to 700 m of moderate- to high-intensity side steps [3], highlighting the relevance of changing direction during side-stepping movements [4,5].

Although COD performance has been considered one of the most important physical qualities in a wide variety of team-sports, including handball [3,6–8], information about COD in this sport is still scarce, with few studies analyzing this skill [1,3], as well as differences between levels and age categories. Better understanding of COD movements would help in the design of training interventions aimed at optimizing player performance and preparation. This is especially important at young ages, when maturation leads to an “adolescent awkwardness”, referring to a temporary disruption in basic motor skills resulting from the early onset of the adolescent growth spurt [9], generally occurring ~6 months prior to peak height velocity (PHV) [10].

In order to assess the strengths and weaknesses of a given player, standardized physical measurements are commonly used to provide useful, and more precise information, to subjective coaching appraisals of player´s performance [11]. In this regard, the inclusion of COD assessments in testing batteries has become very popular in the last few years, including a wide variety of tests related to different sports [12]. This was due, in part, to the previous research suggesting that acceleration, maximum speed, and COD may be considered as separate abilities (14,15). Although COD drills differ in terms of complexity and duration, one of the most adequate protocol for intermittent sports is the ‘505 COD test’, in which the athlete turns once to sprint 5 m back to the starting line [12]. More recently, the “COD deficit” calculation has been introduced [13], by comparing the time or velocity in the 505 test with a linear sprint over an equivalent distance (e.g., 10-m time vs. 505 time). In practical terms, the COD deficit has been used as an additional method for assessing COD ability in different team sports, including handball [3], being considered as an indicator of the athlete’s efficiency in changing direction (i.e., lower COD deficits represents higher efficiencies to change direction) [14]. As such, the evaluation of COD performance in conjunction with COD deficit calculation seems to be a useful and consistent reference for coaches and researchers, especially in young team sport players, in order to develop more efficient strategies to improve COD ability. This study aimed to compare the differences in sprint, jump, COD performance, and COD deficit between under-13 (U13) and under-15 (U15) male handball players.

## Method

### Experimental approach to the problem

This cross-sectional comparative study was conducted to compare the physical performance of two different age-categories of handball players (U15 and U13), from a top-level Spanish handball club. Testing sessions were undertaken between 16:30 and 19:00 hours, and players were assessed at their regular training facility. The testing took place in an indoor court (temperature, 22.3–24.4°C; relative humidity, 54.4–61.0%; Kestrel 4000 Pocket Weather Tracker, Nielsen Kellerman, Boothwyn, PA). The young athletes were previously familiarized with procedures and assessment routines, which were completed on the same day. In this regard, four days before the measurements, all players participated in a pilot session. The data collected in this session were used to calculate the between-day intraclass correlation coefficients (ICCs). Subjects were required to refrain from any intense physical workout for 24 h before the tests and to be in a fasting state for at least 2 h. The order of assessments was as follows: (1) anthropometric measurements, (2) countermovement jump (CMJ), (3) triple leg-hop for distance, (4) linear sprint test, and (5) modified 505 COD test. Prior to the measurements, athletes performed a standardized warm-up, consisting of jump rope activation, general dynamic mobility, multi-directional accelerations, and submaximal jump attempts.

### Participants

One hundred and nineteen young male handball players took part in this study. For the purposes of the present study, players were grouped into two age groups: U13 (n = 82; age: 13.0 ± 0.6 years; height: 162.3 ± 7.8 cm; body mass: 54.7 ± 11.7 kg) and U15 (n = 37; age: 14.8 ± 0.6 years; height: 170.8 ± 8.3 cm; body mass: 63.3 ± 10.9 kg). Subjects were part of the youth categories of Abanca Ademar León handball club and were selected by their respective coaching staff. All players participated in an average of 540 ± 10.1 min of combined handball and physical training per week and had a training background of 5.5 ± 2.8 years. Table 1 shows a typical weekly training content for the different age-categories. None of the players reported a history of any orthopedic problems or limitations during the previous 12 months.

**Table 1:** Typical weekly training content for the different age-categories of handball players.

Category	Monday	Tuesday	Wednesday	Thursday	Friday	Saturday
**U13** (n = 82)	Tec/Tac 90’	NWU 20’	Tec/Tac 60’	NWU 20’	Tec/Tac 60–90’	FM 50’
		Tec/Tac 60’		Tec/Tac 60’		
**U15** (n = 37)	S/PT 60’	S/PT 60’	Tec/Tac 90–120’	S/PT 60’	Tec/Tac 90’	FM 60’
	Tec/Tac 90’	Tec/Tac 90’		Tec/Tac 60’		

Tec/Tac: technical and tactical training; NWU: Neuromuscular warm-up (e.g., preventive exercises; plyometrics, speed/agility drills); S/PT: strength and power training; FM: friendly match.

Before taking part in the study, subjects and their parents/guardians were fully informed about the protocol and provided their written informed consent. The Institutional Ethics committee (Universidad de León; ref: ETICA-ULE-012-2020) approved the procedures in accordance with the latest version of the Declaration of Helsinki.

### Anthropometric measurements and maturity status

Body height was measured using a fixed stadiometer (± 0.1 cm; Holtain Ltd., Crosswell, UK), sitting height with a purpose-built table (± 0.1 cm; Holtain Ltd., Crosswell, UK), and body mass with a digital scale (± 0.1 kg; ADE Electronic Column Scales, Hamburg, Germany) [15]. Pubertal timing was estimated according to the maturity offset method, as previously described [16]. The age of peak linear growth (age at PHV–APHV-) is an indicator of somatic maturity representing the time of maximum growth in stature during adolescence [17]. Maturity level (in years) resulted from subtracting the chronological age at the time of measurement from the chronological peak velocity age. Thus, a maturity offset (MO) of -1.0 indicates that the player was measured 1 year before his PHV, a MO of 0 indicates that the player was measured at the time of his PHV, and a MO of +1.0 indicates that the participant was measured 1 year after his PHV [18].

### Countermovement jump (CMJ) test

A CMJ without arm swing was performed on a photocell mat (SportJump System Pro, DSD Sport system, Spain) according to the procedures described by Bosco et al. (11). Each player performed two maximal jump trials interspersed with 45 s of passive recovery. Subsequently, the average jump height was calculated and considered for analysis. The ICC of the CMJ was 0.96.

### Triple leg-hop for distance

The triple leg-hop test requires participants to perform 3 consecutive hops on the same leg aiming for maximum distance [19]. The toes of the participants were positioned immediately behind the zero mark of the measuring tape, and the distance covered was measured as the distance (in m) from the zero mark to the point their heels touched the ground following the third hop. To be considered a valid attempt, players had to maintain the balance on the tested foot for at least two seconds, before touching the ground with the non-tested foot. Each player performed two attempts with each leg, interspersed with 45 s of passive recovery. Next, the average jump distance was calculated and used for analysis. The participant’s dominant limb was defined as the preferred stance leg used when the participant kicked a ball as far as possible [19]. The ICC of the test ranged from 0.92 to 0.97.

### Linear sprint test

Time during a 20-m sprint (with 5 and 10 m split times) in a straight line was measured by means of single beam photocell gates placed 1.0 m above the ground level (DSD Sport system, Spain). Each sprint was initiated 1.0 m behind the first photocell gate, which then started a digital timer. Players started the sprint test in a standing position with their preferred foot behind the starting line, followed by accelerating forward at maximal effort until they passed the last photocell gate placed at 20 m. Each player performed two maximal 20-m sprints with at least 2 min of passive recovery in between the two trials [20]. The average performance was calculated. The ICC for this test was 0.96.

### Modified 505 COD test

The ability of athletes to perform a single and rapid 180° directional change over five meters was measured using a modified version (stationary start) of the 505 test [21]. Players started in a standing position with their preferred foot behind the starting line, followed by accelerating forward at maximal effort until reaching a line placed at 5 m. Two trials pivoting on both left and right feet were completed and the average time computed to the nearest 0.01 s (DSD Sport system, Spain) was used for analysis. Two minutes of rest was allowed between trials. The ICC was 0.92. The COD deficit was calculated using the formula: *COD deficit = (modified 505 test time– 10-m test time)* [13].

### Statistical analyses

Data are presented as means ± standard deviations. The statistical analyses were performed using the SPSS^®^ software package version 22.0. (SPSS, Inc., Chicago, IL, USA). Data normality was confirmed using the Kolmogorov-Smirnov test. The differences in age, APHV, MO, height, and weight between the age categories were assessed using an independent t-test. The differences in the physical fitness between U13 and U15 players were analyzed using a univariate analysis with MO as covariate. The statistical significance level was set at *P* < 0.05. Effect sizes (ES) were calculated to estimate the magnitude of differences in the tested variables and interpreted using the following thresholds: <0.2, trivial; ≥0.2, small; ≥0.6, moderate; ≥1.2, large; ≥2.0, very large and; ≥4.0, almost perfect [22].

## Results

**Fig 1:**
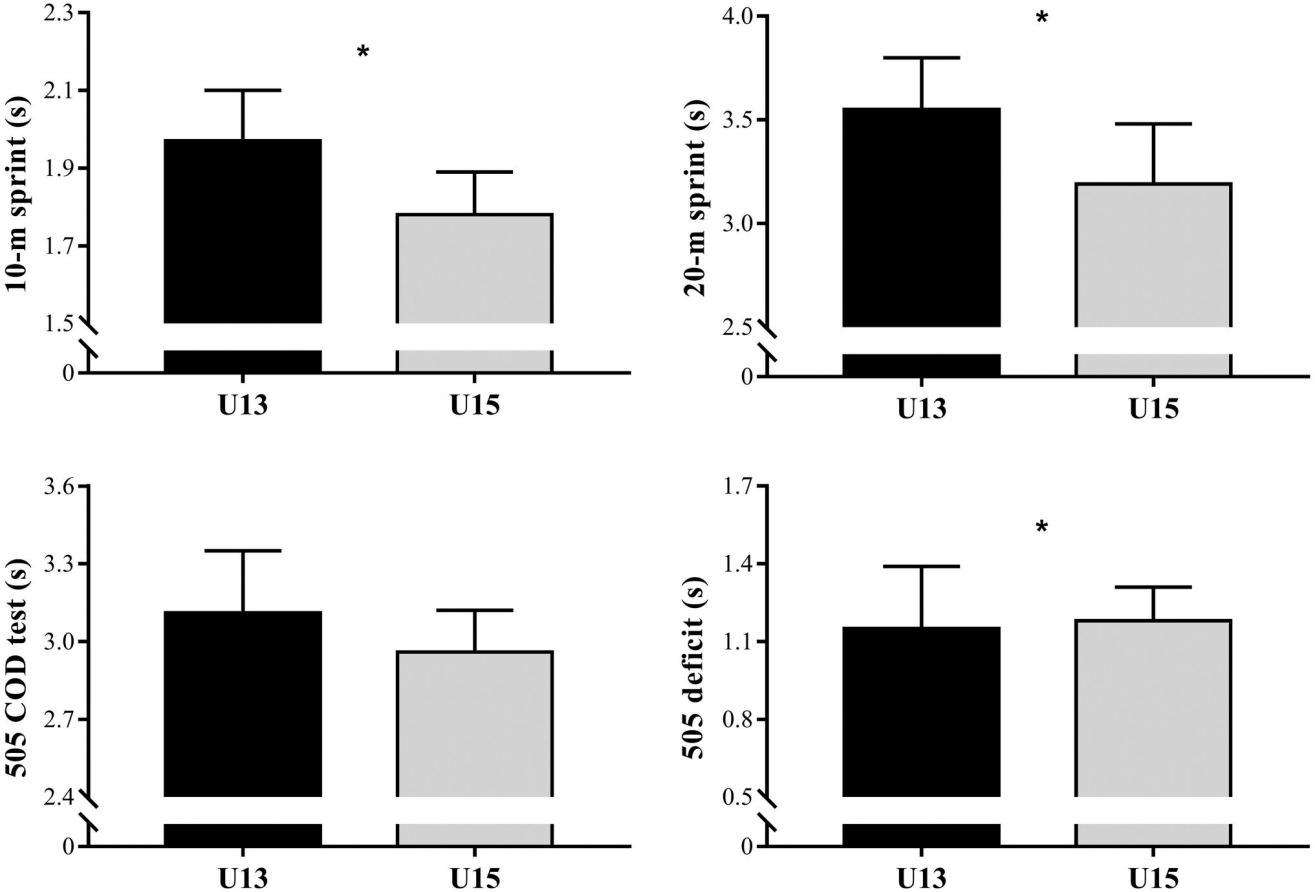
Comparisons of 10- and 20-m linear sprint and modified 505 COD test times, and 505 deficit between under-13 and under-15 handball players; *P< 0.05, considering distance from maturity offset as covariate.

Moderate to very large differences (*P* < 0.05) in age, APHV, MO, height, and weight were observed between the age categories (Table 2). Table 3 shows the comparisons of the CMJ and triple leg-hop test between U13 and U15 players. The U15 players demonstrated higher performances in all jump tests than the U13 players (*P* < 0.05, considering MO as covariate). Fig 1 depicts the comparisons of 10- and 20-m linear sprints, 505 COD test, and 505 deficit between U13 and U15 players. The U15 athletes demonstrated lower sprint times in 10- (ES = 0.84) and 20-m (ES = 0.51) and a higher 505 deficit (ES = 0.38) than the U13 players (*P* < 0.05, considering MO as covariate). No significant differences were observed for the 505 COD test between U13 and U15 groups (ES = 0.12; *P* > 0.05, considering MO as covariate).

**Table 2:** Comparison of age, maturity status, height, and weight between handball players of distinct age categories.

	Under 13	Under 15	Effect Sizes (*rating*)
Age (years)	13.0 ± 0.6	14.8 ± 0.6*	3.18 (*Very* l*arge*)
APHV (years)	14.0 ± 0.6	14.4 ± 0.6*	0.67 (*Moderate*)
MO (years)	-0.97 ± 0.74	0.44 ± 0.84*	1.89 (*Large*)
Height (cm)	162.3 ± 7.8	170.8 ± 8.3*	1.09 (*Moderate*)
Weight (kg)	54.7 ± 11.7	63.3 ± 10.9*	0.73 (*Moderate*)

APHV: age at peak height velocity; MO: maturity offset;

*P < 0.05.

**Table 3:** Comparison of countermovement jump height (CMJ), and triple leg-hop test distance between under 13 and under 15 handball players.

	Under 13	Under 15	Effect Sizes (*rating*)
CMJ (cm)	23.8 ± 4.9	32.3 ± 6.8*	1.00 (*Moderate*)
triple leg-hop test (m)	4.49 ± 0.61	5.77 ± 0.95*	1.04 (*Moderate*)

*P < 0.05 considering distance from maturity offset as covariate.

## Discussion

The aims of the present study were to examine and compare the differences in linear sprint ability, COD performance, and COD deficit between U13 and U15 male handball players. To the best of our knowledge, this is the first study to analyze the COD performance in two age groups of young handball players. Overall, our results showed that the U15 athletes performed better than the U13 players in both jump (e.g., CMJ and triple leg-hop test; differences ranging from 2.6 to 36%) and sprint tests (e.g., differences of ~10%). Regarding COD performance, U15 players also presented a significantly higher deficit (e.g., + 2.6%), although no significant differences were observed for the 505 COD time between groups. These data may be of great interest and have important implications for practitioners involved in the development and training of young handball players.

As expected, U15 players outperformed the younger players (U13) in all performance measurements, which is consistent to previous research describing youth athletes from other team sports (e.g., soccer and rugby) [23,24]. These discrepancies are probably related to the significant differences observed in the maturational status and, hence, in anthropometric variables (e.g., height and weight), favoring the U15 players. In this regard, it has been reported that a more advanced maturational status may lead to greater pubertal gains in height, weight, absolute, and relative muscle mass and, thus, to superior levels of strength, power, speed, agility, and endurance, at both muscular and cardiorespiratory levels [25]. This enhanced capacity to apply force and generate power in the U15 players certainly increases their potential to sprint faster and jump higher, as strength and power parameters have been consistently shown to be strong predictors of these explosive capabilities [26,27].

When analyzing the COD test considering DPHV as the covariate, results indicated no significant differences between groups (e.g., 3.11 ± 0.24 s [U13] vs. 2.96 ± 0.16 s [U15]). These findings are supported by previous studies, showing similar COD performance in different age groups of young athletes (e.g., soccer, rugby, and tennis) [23,24,28]. The lack of differences in the COD speed could be explained by different factors. For example, there is a disproportional growth and disruption of motor coordination in complex motor coordination tasks (e.g., agility) at the ages around and after the PHV [29], a time-point corresponding to a phenomenon so-called “adolescent awkwardness” [23]. Moreover, the superior volume of concurrent training practices observed in the U15 group (Table 1) could have compromised their COD neuromuscular performance, as suggested by previous research [30,31]. In line with these studies, and based on the data about the weekly training reported herein, it seems that the higher amount of technical and tactical training in the regular schedule of U15 players, typically characterized by aerobic-oriented training methods (e.g., small-sided games and technical drills), may be responsible for the meaningful impairments detected in some specific speed-related qualities, such as the COD ability [30]. Thus, it can be recommended to supplement the technical-tactical workouts with training strategies specifically designed to enhance COD performance.

The U15 athletes demonstrated higher COD deficit (e.g., 1.18 ± 0.13%) than the U13 players (i.e., 1.15 ± 0.24%; considering MO as covariate). These data are in agreement with previous studies in team-sport athletes (comprising both professional and youth athletes, including Olympic handball players), which showed that faster and more powerful athletes tend to present higher COD deficits (when compared to their slower and weaker peers) [14,32,33]. The higher COD deficit in the U15 group could be related to the fact that faster (and heavier) athletes possibly have greater “inertia” and, therefore, need to apply higher breaking forces over longer ground contact times [14], not being capable of handling these forces efficiently. As a result, and based on recent research [14,34,35], the inclusion of training practices combining not only physical elements, but also cognitive and technical skills (e.g., unanticipated COD tasks) more related to sport specificity, may increase the magnitude and efficiency of external training loads, which could be useful for these athletes [36]. Moreover, the use of training methods focused on increasing eccentric strength is suggested to improve the ability to tolerate high braking forces during deceleration prior to directional changes.

We have to acknowledge some limitations in the current study, including the lack of a larger sample of young athletes, especially in the U13 group, or having more distinct age groups to examine the evolution of these parameters throughout the players’ development. Another limitation is related to the fact that 505 test involves one single 180° COD and, thus, may not be represent the actual capability of players to successively change direction while playing. The implementation of other drills, using different cutting maneuvers (i.e., 45° to 90°), closer to the movement patterns observed in handball (e.g., forward and backward run, side stepping, multiple changes of directions) can be also useful to evaluate these athletes. What it is clear here is that, due to the importance of COD performance in handball, coaches and technical staff should gradually include specific training practices for improving this complex physical quality from the younger categories. Training sessions comprising consecutive accelerations and decelerations, planned and unplanned agility drills, and technique exercises are recommended to properly develop COD performance in young handball players during their specialization years.

## Conclusions

In conclusion, in youth handball players, jump (e.g., CMJ and triple leg-hop test) and sprint (e.g., 10- and 20-m) performance was superior in U15 than U13 players; nonetheless, when it comes to COD performance, there were no significant differences between players. In contrast, U15 players presented a significantly higher COD deficit than the U13 players. These findings have important practical applications for coaches and sport scientists when training or testing young handball players. In general, it appears that, during the transition from pre- to post-puberty, young handball players should focus on transferring their progressive increases in strength, speed, and power capacities to COD performance. As such, more mixed training strategies (e.g., resisted sprints, horizontally directed power exercises, and eccentric strength training) [7,8] may be necessary to improve this complex physical skill, also combined with technically-oriented exercises to improve efficiency during COD maneuvers [34]. Further studies are warranted to investigate and define the best training approaches and content to effectively develop speed-related performance in young handball players.

## Supporting information

S1 Data (XLS)
